# Co-designing an intervention using the COM-B model to change an eating behaviour in people living with achalasia

**DOI:** 10.3389/fmed.2024.1216209

**Published:** 2024-04-30

**Authors:** Melika Kalantari, Amelia Hollywood, Rosemary Lim, Majid Hashemi

**Affiliations:** ^1^School of Pharmacy, University of Reading, Reading, United Kingdom; ^2^University College London Hospitals NHS Foundation Trust, London, United Kingdom

**Keywords:** achalasia, co-design, COM-B model, intervention, behaviour change, eating behaviour, chronic condition, rare condition

## Abstract

**Background:**

Achalasia is a rare motility disorder affecting the oesophagus, leading to difficulties with eating and drinking. Participants in previous studies reported that they needed more social, clinical and behavioural support in the long-term management of achalasia. This study, therefore aimed to 1) identify the most challenging eating behaviour for people living with achalasia and 2) co-design a behaviour change intervention to help address the challenges they experience.

**Methods:**

This study used a qualitative approach involving online focus groups. The COM-B model was the theoretical framework, with behaviour change techniques (BCTs) as the active ingredients that target a mixture of capability, opportunity and/or motivation. Three focus groups were undertaken to obtain a range of input from different people living with achalasia. Participants in this study identified the target behaviour, prioritised the different BCTs which most resonated with them to design an intervention and decided on the mode of delivery. The research team analysed the techniques that helped participants with their eating behaviour using the COM-B model as a framework to create the intervention.

**Results:**

The 24 participants in this study identified “eating in a social setting” as the target behaviour for the intervention. A workbook that can be personalised by the individual was the most suitable intervention. The workbook structure aligns with the constructs of the COM-B model. It includes reflection, activities and goal-setting sections based on what was indicated to be useful for the majority of the participants. Key techniques to overcome the challenges with eating in a social setting included social support, regulation to reduce negative emotions, goals and planning.

**Conclusion:**

Using a focus group approach with the COM-B model as the theoretical framework, the participants in this study developed an intervention to support people living with achalasia. In order to achieve long-term behaviour change, engagement with a personalised workbook could facilitate eating in a social setting. Future work will need to pilot the workbook to ensure it can support people to improve their quality of life and complement the ongoing care they receive from health services.

## Background

Achalasia is a rare motility disorder affecting the oesophagus. This condition can start at any time of life but is more common in middle-aged or older adults (1). It is equally prevalent in males and females, with an overall incidence of 1.63 cases per 100,000 people (2, 3). The underlying causes of achalasia are unknown (1). Characteristic features of the condition include a non-relaxing sphincter, weak or absent oesophageal peristalsis and simultaneous or poorly coordinated contraction, leading to an outflow obstruction at the level of the lower oesophageal sphincter (LOS) (1). The presence of these features leads to difficulty in swallowing liquids and solid food, and a variety of other associated symptoms such as painful spasms, regurgitation, heartburn and choking (1). Despite it being a disabling condition, about 20–50% of cases are initially misdiagnosed, with patients given an alternative diagnosis such as gastro-oesophageal reflux disease (GORD) or hiatus hernia (4). Treatment for achalasia is often delayed due to a lack of diagnosis, and even the most effective treatments are unlikely to be curative (2). Therefore, in a large proportion of patients, the initial treatment is either delayed or inappropriate and ineffective (5). Moreover, as the cause of achalasia is unknown, treatment has focused on alleviating the symptoms and their consequences. There are different treatment options available for achalasia, including medication (i.e., muscle relaxants), Botox injections, pneumatic dilatation (PD), surgical interventions, such as laparoscopic Heller myotomy (LHM) and peroral endoscopic myotomy (POEM), and non-medical interventions, such as behavioural changes. As treatment options do not provide a definitive cure, it is, therefore, critical that people with achalasia learn to self-manage their symptoms (to some degree).

There are several behaviours that can trigger symptoms in achalasia. Eating behaviours were one of the main problems that were reported by participants in a previous study (6). In this study, people living with achalasia were interviewed to explore their experiences and management of the condition. Different eating behaviours, such as the type of food, time of the meals and unhealthy eating, such as grazing, were identified as behaviours that exacerbate their symptoms in the ongoing management of achalasia (6). This highlights that people living with this chronic condition need to make daily decisions about their illnesses. This ownership of managing a chronic condition introduces a new paradigm which involves collaborative care and self-management education. This approach supports people to have the best possible quality of life with their chronic condition by adopting a new paradigm of care. Self-management education teaches problem-solving skills. Self-efficacy, which is a central concept in self-management, gives the patient the confidence to carry out the necessary behaviours in order to reach the desired goal (7). According to the study carried out by Michie et al. (8), behaviour changes are facilitated by the 93 active ingredients of the behaviour change technique taxonomy. These techniques include goal setting and provision of instructions (9). The behaviour change wheel (BCW) is a framework that promotes a systematic method of intervention development (9). At the core of the BCW framework is a theoretical model called the COM-B model. Based on the COM-B model of behaviour by Michie et al. (9), individuals should have the physical and psychological capability, opportunity and motivation in order to perform a behaviour.

The current study utilised the COM-B model and Theoretical Domains Framework (TDF) to systematically develop the intervention and embrace a participatory co-design approach, involving individuals who are experts due to their firsthand experience with the condition. The aim of the study was to co-design a behaviour change intervention tailored to address the specific challenges experienced by people living with achalasia.

## Methods

### Design

This study involved a co-design approach to design an intervention with and for people living with achalasia. This approach is beneficial in order to get first-hand information from people living with the condition and use their experiences to develop an intervention. It also allows people involved in the co-design process to reflect on their experiences of a particular subject and work together to identify improvement priorities, implementing changes, and then jointly reflecting on their achievements (10). A favourable ethical opinion was granted through the University of Reading School of Chemistry, Food and Pharmacy Research Ethics Committee (SREC 40/2020).

### Procedure

The study design for this research was focus groups. A focus group is ideal when discussing a topic with a selected group of people, as it helps to obtain several perspectives about the same topic. Homogeneous groups provide a relatively safe place for participants to share their experiences and, in the case of medical education research, mitigate the power imbalance between researcher and researched by utilising the naturally existent peer group (11). This design allows the researcher to explore the degree of consensus on a given topic and gather a larger amount of information in a shorter period of time (12).

**Figure 1:**
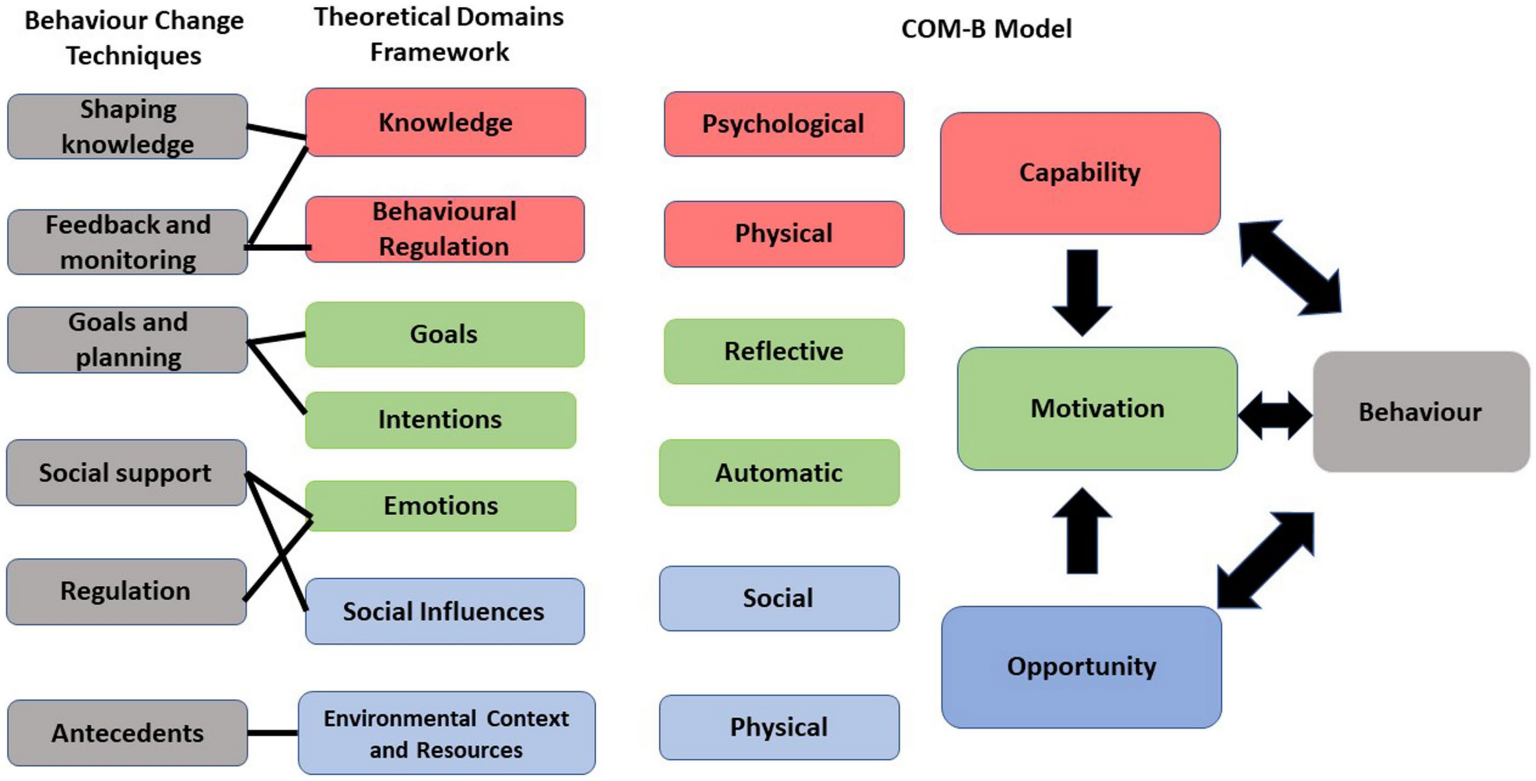
Link between BCTs, TDF, and COM-B model.

The focus groups were conducted online using Microsoft Teams, which is a videoconferencing platform. The aim of the first focus group was to discuss eating behaviours that related to achalasia, identify the target behaviour for the intervention and discuss techniques to change that specific eating behaviour. Participants in the first focus group were presented with a list of behaviours [identified from previous research (6)] and were asked to identify the most challenging one. The eating behaviours included the time of the meals, types of food, food avoidance, grazing, binge-eating, eating when stressed and eating in private and/or public. The second focus group aimed to co-design an intervention targeting the identified eating behaviour using the COM-B model as the theoretical framework. The third focus group verified the co-designed intervention and determined the best delivery method for that intervention. The research materials, such as a presentation for each session, were prepared by the research team before each focus group. The TDF was used to create a topic guide with prompt questions. The TDF mapped onto the overarching COM-B paradigm, which provided structure to the focus groups. Figure 1 shows the link between BCT, TDF and the COM-B. The presentation included a background of the targeted behaviour and each element of the COM-B model with examples in order to help participants to discuss the prioritised eating behaviour, which was the aim of the study. The presentation was used as a topic guide to facilitate discussions in the focus group. Participants were asked to discuss the techniques presented to them within each component of the model and discuss what might work best for them in order to change the targeted eating behaviour. Participants in the final session confirmed the researchers’ interpretations from the other two sessions and decided on the delivery method of the co-designed intervention. After the focus groups, details of the co-designed intervention and its delivery method were discussed within the research team. The information provided in the focus groups was categorised into different elements of the COM-B model and refined within the research team.

### Recruitment

A sample of 24 people living with achalasia took part in this study. The sample size was based on focus groups typically involving 4 to 8 participants in each session (this study involved 3 focus groups) (13). Moreover, previous qualitative research in similar contexts suggested that a sample size of approximately 24 participants would likely be sufficient to reach data saturation, where no new themes or insights were provided by additional participants (14).

The researcher emailed the recruitment materials, which included an information sheet and consent form, to the administrator of the support group. The Achalasia Support Group is affiliated with Achalasia Action, which is an independent charity supporting people living with achalasia in the United Kingdom (UK) (15). The administrator distributed the study materials to potential participants using the support group mailing list. This is a national group, with over 400 members, based in London and run by members of the public living with achalasia. People usually find and join this group through searching on the Internet and word of mouth. Convenience sampling was the strategy used to recruit participants based on their willingness and availability to take part. All members of Achalasia Action were sent the recruitment materials and participants were asked to contact the researcher (MK) if they were interested in taking part in a focus group. They were then asked to complete an online consent form followed by completing an online demographic questionnaire asking their gender, age, living status (living alone or co-habiting) and the type of medical interventions they had previously received for achalasia. Once the desired sample size was achieved, recruitment to the study stopped. The inclusion criteria for this study were as follows: anyone living in the UK, aged 18 years or over, with a confirmed diagnosis of achalasia, who consented to participate, was able to join one of the online sessions and spoke and understood English. Participants were only allowed to join one focus group, and they were signed up to their preferred time slot using a Doodle poll, which is a web-based scheduling tool.

### Data collection

This study involved three in-depth, semi-structured focus groups. Participants in the current study were allocated to one of the three focus groups based on their availability. Focus groups were held over 4 months, with the first two being 2 weeks apart from November 2020 and the last one held in February 2021. We aimed for a maximum of eight participants in each focus group. The aim of the study, the structure of the focus groups and the topics of discussion were stated at the beginning of each online session. The ground rules were explicitly stated and included confidentiality, raising concerns during sessions and the option to withdraw from participation. The online focus groups lasted around 2 h and were recorded for accuracy check purposes. Two female researchers facilitated each session. Session 1 was run by two pharmacist researchers, and a pharmacist and a health psychologist ran the following 2 sessions.

### Focus group 1

Participants in the first focus group were presented with a list of eating behaviours derived from previous research exploring people’s experiences living with achalasia (6). Participants were asked to prioritise those eating behaviours from the most to least challenging. After reaching a consensus in the first focus group, participants started discussing their ideas and opinions about the techniques that could facilitate a change in behaviour.

### Focus group 2

In the second focus group, participants were asked to discuss and co-design an intervention using the COM-B model. Those BCTs, which were shown to be effective in other behavioural change studies (16), were presented to the participants. Participants were asked to discuss and comment on the techniques and share their experiences in order to tackle the challenges with the chosen target behaviour; this included eating in a new environment or eating with different people. The researchers provided examples and prompts, such as making a food diary or educating friends and family, to participants and asked them to discuss other similar techniques that can help them change their eating behaviour. Participants were asked to add more examples of techniques that map onto each element of the COM-B Model.

### Focus group 3

In the final focus group, participants were presented with the findings from the previous focus group sessions. They were asked to confirm whether the proposed techniques would be suitable for them as well. They were also asked to discuss the delivery method of the co-designed intervention, i.e., an online platform or printed materials such as an evolving workbook that can be personalised based on individual’s needs.

Two researchers were present at each focus group to ensure all the points that were raised were documented. At the end of each focus group, the researchers recapped discussion points and ensured everyone agreed with the accuracy of the summary of information from what was shared in the online session. The sessions were recorded and used for accuracy check purposes only. Participants’ discussions in the online focus groups were structured using the COM-B model as the theoretical framework and formed the basis of the developed intervention.

### Data analysis

The collected data were discussed within the research team after each online focus group. This included discussing and refining the main points discussed in the focus groups using the COM-B model. The refined data, such as the suggested techniques, were used as discussion points for the next focus group and helped the researcher to prepare the presentation for the next focus group. A presentation was prepared before each online focus group based on the information provided in the previous online session. The techniques discussed in each session were categorised according to the COM-B model components The suggested techniques, such as ways to manage expectations (feedback and monitoring) or getting support from a buddy (Social support), were in line with the elements of the COM-B model. Quotes were taken from the recordings to highlight the techniques discussed within each element of the COM-B model. Through this analysis, the techniques were finalised and validated by the participants in the final session, where participants were asked to confirm whether the techniques would work for them to change the target eating behaviour. Consensus was confirmed when all participants in the final session agreed on the components and co-designed intervention from the previous focus groups and no more new ideas were discussed.

## Results

The results are organised according to the constructs of the COM-B model, which include Capability, Opportunity and Motivation, which are necessary conditions for the desired behaviour change. Participants provided rich insights into living with achalasia for each element of the COM-B model. They identified the target behaviour, which was eating in a social setting, then discussed strategies that were implemented to facilitate behaviour change. Participants in this study agreed that the co-designed intervention to help them change the target eating behaviour would be an evolving workbook that can be tailored and personalised based on the needs of each individual living with achalasia.

### Sample

This study included 24 participants living with achalasia. The age range of participants who completed the questionnaire was from 29 to 77 years (mean 53). All participants reported trying to change their eating behaviour in the past. Five participants reported managing their symptoms with no medical interventions. Fifteen participants had one medical procedure (HM *n* = 10; PD *n* = 3; Botox *n* = 2) and four participants underwent multiple medical treatments (HM and PD *n* = 2; HM and PD *n* = 1; HM and POEM *n* = 1; PD and Botox *n* = 1). All participants reported having symptoms of achalasia even after medical treatment. Eight participants attended the first focus group, eight in the second focus group and eight in the last focus group. Table 1 shows the demographic details of the participants.

**Table 1:** Participant demographics.

Participant demographics	All participants (*n* = 24)
Gender	Male *n* = 3 (12.5%) Female *n* = 21 (87.5)
Age (years)	Median (SD) 52 (14.19) Range 29–77
Ethnicity	White *n* = 23 (96%) Asian *n* = 1 (4%)
Living status	Living alone *n* = 4 (16%) Co-habiting *n* = 20 (84%)
Employment status	Full-time *n* = 8(33%) Part-time *n* = 7 (29%) Retired *n* = 8 (33%) Not working *n* = 1 (4%)
Attempted to change eating behaviours in the past	*n* = 24 (100%)

### Behaviour

The target behaviour for this study was “eating in a social setting” which was prioritised and chosen by participants amongst a list of behaviours identified from previous research (6). Eating in a social setting was identified as the most challenging eating behaviour as it impacted participants’ quality of life the most. Table 2 outlines a list of techniques reported by participants that can be performed to change the target behaviour.

**Table 2:** Intervention type based on different components of the COM-B model.

COM-B components	Techniques suggested by participants (Intervention function: behaviour change technique (BCT): example)
Physical capability	Training: exposure: building the stamina to tolerate new environments
Psychological capability	Education: instruction on how to perform a behaviour: increasing knowledge about the menu or the restaurant before eating in an unfamiliar setting
Physical opportunity	Enablement: reduce negative emotions: Trying to reduce negative emotions when eating in an unfamiliar setting through stress management skills
Social opportunity	Modeling: practical social support: using techniques used by other people to increase confidence when eating in an unfamiliar setting
Reflective motivation	Incentivisation: self-reward: set a reward after enjoying and embracing eating in an unfamiliar setting
Automatic motivation	Persuasion: instruction on how to perform a behaviour: speaking to others living with achalasia to induce positive feeling about eating in an unfamiliar setting

### Capability

Capability can be described as the individual’s psychological and physical capacity to engage in the activity (17). Both physical and psychological capability can be improved through interventions. It is crucial to understand why and how to make the change along with having the capacity and skills to sustain it. Participants were presented with a few of the techniques within the capability element and were asked to discuss potential activities that could be incorporated in the intervention. The two techniques that were presented to the participants were feedback and monitoring; and shaping knowledge as these were the techniques shown to be effective in other studies for changing behaviours (17). When participants were presented with the capability component, they discussed how physical and mental ability to change eating behaviour in public is important. This includes the need for knowledge, such as familiarising themselves with the menu and the stamina to tolerate the new environment to perform a behaviour change. The quotes below demonstrate examples of the behaviour change techniques discussed by participants in the focus groups.

#### Feedback and monitoring

The following quote provides insight into how to perform and gain feedback on the target behaviour:

*“My view on this is before you go to a restaurant, to be in a mindset, because you want to enjoy your meal, you do not want to be anxious; you have to live with your condition. So, I feel that you have to compose yourself, meditate, breathe deeply, possibly have a little bit to eat beforehand so that you are not incredibly hungry, so you do not go to the restaurant with low sugar levels, being anxious, being worried because you are there to enjoy your company.” (focus group 2)*

#### Shaping knowledge

A participant mentioned how looking at the menu prior to eating out can give them the knowledge of what they can eat when they are in an unfamiliar social setting.

*“I have to admit my first thing when I look at a menu and as others have said as well, I will if it’s online beforehand I will always do that as well and I tend to go through menus not with what I would like to eat, but I look at all the sort of options and think well, cannot have that! Cannot have that! Cannot have that! and then I’ll look and think oh, out of the three things out of 20, which one could I possibly have?” (focus group 2)*

### Opportunity

Opportunity can be described as all the factors that lie outside the individual that make the behaviour possible (17). These are factors in the environment that encourage or discourage achieving behaviour change. The two techniques that were presented to the participants in this study were antecedents and regulation as they were shown to be effective in other research (18). These include restructuring the physical/social environment (antecedents) and reducing negative emotions (regulation). Participants discussed finding ways to overcome barriers such as reducing stress or changing the environment and creating a more suitable space to perform the desired behaviour.

#### Restructuring the physical/social environment

One participant mentioned how restructuring the environment would put her mind at ease and she could go back to her eating after calming herself down.

*“Now I abandoned the meal completely. If I’m feeling hot and flustered, I’ll go and sit in a cool place, sometimes I have to take up the meal with me, if that’s not going to work, the meals put aside, I sit down, collect my thoughts, and perhaps an hour later I’ll have my meal.” (focus group 3)*

#### Reducing negative emotions

A participant mentioned how negative emotions such as stress could cause problems while eating out.

*“I’m just thinking, one thing when we were talking about negative emotions, never eat when you are really stressed, or if you have had a major disagreement with someone, it will bounce back on you, literally. You’ve got to wait to calm down. Deep breathing, fresh air, cool, literally cool myself physically, to calm down before I eat.” (focus group 3)*

### Motivation

Motivation is described as “all the brain processes that energise and direct behaviour,” not just goals and conscious decision-making (16). The techniques within the motivation element include; goals and planning, and social support. These were the techniques that were presented to the participants in this study as they were shown to be effective in other research (16). The reflective and automatic motivation were discussed by the participants. Developing a plan to promote the desired behaviour and social support were key points that were discussed in the focus groups.

#### Goals and planning

One participant mentioned how having a plan will help her eating in a social setting.

*“I just always have a plan of action when I get to the restaurant, and I always talk to the staff there about what the food actually is, I’ll make sure that it might say something on the menu and then I find well, after seeing someone else with it, I think that’s very dry, and it does not seem to be any sort of gravy. So, I mention all these sorts of things, like vegetables can be whole, but they gotta be mashable that that sort of texture and need a sauce of some sort. I just talk to them and that’s that seems to help.” (focus group 1)*

#### Social support

A participant described how an online support group has been helpful since she got her diagnosis.

*“So I’ve really had no support from the hospital and from the consultants at all, and everything I know and everything I’ve done is being based on advice from people and I completely agree with you I see some people saying that they can eat something, and I could not go near it and then other people suggesting something else that works for me. So, I think the online support has been good because you are getting a really broad range of very different people and you can pick and choose the advice that works for you.” (focus group 1)*

### Delivery method

Participants in the final session were in agreement with the techniques suggested in the two previous sessions and no new elements were discussed. They discussed different delivery methods for the co-designed intervention. Participants discussed different tools which can be used as an aid to prepare themselves for eating in a social setting. Hence, they already have access to an information pack through the support group, which includes general information about achalasia; they believe having an evolving workbook to use when eating in a social setting can be very beneficial. They wanted the co-designed intervention to be editable so they could personalise it based on their individual need. Participants agreed on having a workbook to address this specific issue which can work well alongside the more general information available to them through the Achalasia Action support group. Table 3 outlines the techniques discussed within each element of COM-B and the activities suggested by the participants.

**Table 3:** Behaviour change techniques suggested by people living with achalasia.

COM-B model constructs	Behaviour change techniques	Examples
Capability	Feedback and monitoring	Reflect on what triggers symptoms when at a restaurant Eat mindfully
	Shaping knowledge	Pre-check restaurant menu online Address dietary restrictions openly with restaurant staff
Opportunity	Restructuring the physical/Social environment	Request a jug of water to help with swallowing Take a break and get fresh air as needed
	Reducing negative emotions	Avoid eating during high-stress periods Use relaxation and posture techniques
Motivation	Goals and planning	Have a plan of action before going to a restaurant Discuss food textures with restaurant staff
	Social support	Access online support group for advice Share experiences with others

### Overview of findings

Participants in this study agreed that an evolving workbook, which includes different sections based on the COM-B model, would be the most suitable intervention for people living with achalasia to facilitate eating in a social setting. Goals and planning, feedback and monitoring, antecedents, shaping knowledge and social support were the techniques highlighted in the focus groups to be included in the workbook to help people living with achalasia change their eating behaviour in a social setting. They also confirmed that the intervention should be a workbook available in hard copy and online.

## Discussion

This is the first study to the authors’ knowledge that used the COM-B model to co-design an intervention to help people living with achalasia change their most challenging eating behaviour. In this study, participants prioritised eating in a social setting as the most challenging eating behaviour, and the COM-B model was used to co-design the behaviour change intervention.

### Capability

This research highlights a lack of information can lead to stress and trigger symptoms when eating in a social setting. Similarly, a lack of information has also been shown to impact capability in relation to making lifestyle changes and adhere to treatment when diagnosed with gestational diabetes mellitus (GDM) (18). Women with GDM needed to be provided with adequate health information, appropriate educational resources and patient-centred counseling to achieve lifestyle changes (18). A lack of information and monitoring in people living with achalasia results in stress and anxiety, which affects eating behaviour. All participants in this study discussed the importance of preparing themselves before eating out, including increasing their knowledge by reading the menu in advance or creating an informative tool to explain their situation to others. The results of this study also show that feedback and monitoring on past events and gaining information can reduce stress when it comes to eating in a public setting, hence reducing the challenges with eating.

### Opportunity

Our findings show that people living with achalasia can reduce their negative emotions by engaging in activities, such as reading the menu before eating in a social setting, and restructuring their physical and social environment, such as taking a break from the dining table. This finding aligns with the study carried out by Carney et al., where researchers used the COM-B model to develop an intervention to promote physical health for young people (16). Environmental factors were shown to be crucial when developing the intervention (19) and a local gym with a safe environment was encouraged rather than exercising outdoor. These findings highlight the importance of the social and physical environment in facilitating behaviour change.

### Motivation

The current study demonstrates that motivation techniques, such as goals and planning and social support, can help people living with achalasia reduce the challenges of eating in a social setting. Existing research suggests that social support may, directly and indirectly, improve self-care behaviours and highlights the importance of interventions that augment patients’ confidence (20). The intervention co-design in this study allows people living with achalasia to access relevant and appropriate resources through the evolving workbook, and increase confidence when eating in a social setting. It will also allow people to plan ahead and reflect on their goals when eating in a social setting.

### Strength and limitations

Our study has several strengths to discuss. The qualitative approach, specifically the use of focus groups, provided a rich platform for capturing the lived experiences of individuals living with achalasia. This approach enabled us to gain valuable insights into their challenges and preferences, which were instrumental in co-designing an effective behaviour change intervention targeted at a specific eating behaviour. Importantly, our recruitment strategy through Achalasia Action, a platform for sharing experiences, facilitated a collaborative environment where participants could share their collective ideas regarding interventions that had been effective or not. The COM-B model provided a theoretical framework to facilitate a structured and evidence-based process for developing the behaviour change intervention. The different elements of the COM-B model, i.e., capability, opportunity and motivation, the TDF and its techniques, along with patient and public involvement were incorporated to strengthen the intervention co-design.

We also recognise limitations in our study. Firstly, our recruitment strategy may have introduced a selection bias, potentially impacting the generalisability of our findings to the broader achalasia patient population. Members of the Achalasia Action support group have access to online resources, support, and interactions with other people living with achalasia. However given the rarity of the condition, we opted for this recruitment approach given its success in previous research (6). Furthermore, our study included a relatively small sample size of 24 participants, which may limit the generalisability of our results. However with the methodology that was employed, the focus group size of eight per session ensured the guided group discussions allowed all participants to share their views and collaborate to develop new ideas. Also the intervention was fully developed by the third session with no new ideas emerging, indicating the number of focus group iterations was sufficient to achieve the study aim. It is important to highlight that there are differences among patients lived experiences with achalasia, including obtaining a diagnosis, receiving different medical treatment and disease duration (6). The variability in patient experiences could impact on the effectiveness of the intervention that has been co-designed. However this issues has been mitigated in the co-design process with participants identifying that the intervention would need to be editable so they could personalise it based on their individual need. The COM-B model would be used consistently for all those receiving the intervention but the reflections, activities and goal-setting that participants engage with can be personalised and tailored to meet their individual need. Future investigations may focus on the potential impact of these variables to gain a more comprehensive understanding of behaviour change in the context of achalasia.

In terms of practical implications, our study underscores the importance of tailoring interventions to the specific needs and challenges of individuals living with chronic conditions such as achalasia. It highlights the potential benefits of co-designing interventions that consider the unique experiences of this population. Moreover, our work emphasises the utility of the COM-B model and the TDF as valuable tools for guiding the development of behaviour change interventions in healthcare settings. It also highlights the importance of considering patients lived experience in healthcare interventions to ensure the variability and specific needs are considered for optimal patient centred care.

### Future research

Future studies should explore the feasibility and effectiveness of the co-designed intervention which is an evolving structured workbook including reflection, activities and goal setting. If feasible, the co-designed intervention can be piloted to all the members of the support group. Additionally, efforts to engage individuals who are not part of support groups or who have not previously attempted interventions could help broaden our understanding and enhance the applicability of such interventions.

## Conclusion

In summary, the study identified the need for an evolving workbook to address challenges related to social eating in people living with achalasia. The theoretical framework and co-design approach facilitated the effective development of a behaviour change intervention that can now be assessed for efficacy. If future research identifies the co-deigned intervention significantly improves quality of life for people living with achalasia, it can be implemented in clinical practice to support patients with the long-term management of this chronic condition.

## Data availability statement

The raw data supporting the conclusions of this article will be made available by the authors, without undue reservation.

## Ethics statement

The studies involving humans were approved by University of Reading School of Chemistry, Food and Pharmacy Research Ethics Committee. The studies were conducted in accordance with the local legislation and institutional requirements. The participants provided their written informed consent to participate in this study.

## Author contributions

MK, AH, RL, and MH made a substantial contribution to the design of the work. MK conducted the focus groups along with AH and RL. MK, AH, and RL extensively discussed how to analyse and interpret the data, discussed the content and the layout of the co-designed workbook. AH and RL contributed to and supervised the analysis of the collected data from the focus groups. All authors contributed to the article and approved the submitted version.
